# Recurrence of Primary Aldosteronism 10 Years After Left Adrenalectomy for Aldosterone-Producing Adenoma: A Case Report

**DOI:** 10.3389/fendo.2021.728595

**Published:** 2021-09-24

**Authors:** Linghui Kong, Jin Zhang, Lei Dong, Jianzhong Xu, Ping-Jin Gao, Ji-Guang Wang, Limin Zhu

**Affiliations:** ^1^ Department of Cardiovascular Medicine, Ruijin Hospital, Shanghai Institute of Hypertension, Shanghai Jiaotong University School of Medicine, Shanghai, China; ^2^ Department of Hypertension, Ruijin Hospital, Shanghai Jiaotong University School of Medicine, Shanghai, China; ^3^ Department of Pathology, Ruijin Hospital, Shanghai Jiaotong University School of Medicine, Shanghai, China

**Keywords:** primary aldosteronism, aldosterone-producing adenoma, adrenalectomy, recurrence, *KCNJ5*

## Abstract

A 29-year-old female patient diagnosed with primary aldosteronism (PA) in 2004 underwent complete adrenalectomy for left aldosterone-producing adenoma (APA) confirmed by hematoxylin and eosin (HE) and CYP11B2 staining. Her hypokalemia was corrected, and her blood pressure (BP) normalized and maintained without medication for 10 years. In 2014, her BP became elevated again, and a recurrence of PA with an adenoma on the right adrenal gland was discovered by computed tomography scan. She underwent partial right adrenalectomy in 2018 due to unsatisfactory BP control with medication and gradually enlarging adenoma. The resected adrenal tissue contained a CYP11B2 staining positive APA. Her BP was then controlled by two drugs. Sanger sequencing of DNA extracted from tissue slices revealed that both left and right adenomas carried the same aldosterone-driver *KCNJ5* gene mutation, but with different nucleotide changes. We suggest that patients who undergo adrenalectomy for APA should be followed up for life.

## Introduction

As 5–10% of hypertensive patients are diagnosed with primary aldosteronism (PA) (1), PA is one of the most common forms of secondary hypertension. While PA is broadly characterized by the high autonomous secretion of aldosterone and suppression of renin activity (PRA), it can further be categorized into the main subtypes of aldosterone-producing adenoma (APA) and bilateral adrenal hyperplasia. Unilateral adrenalectomy is recommended for the treatment of APA, as this method of intervention achieved biochemical and clinical success in approximately 94% and 17%–62% patients, respectively (2). To date, few reports have considered the recurrence of PA after unilateral adrenalectomy with complete clinical and biochemical success (3, 4).

With the development of exome sequencing analysis, mutations in *KCNJ5, ATP1A1, ATP2B3, CACNA1D, CACNA1H, CLCN2*, and *CTNNB1* in APA has been reported successively (5–12). Majority of these mutations cause abnormal adrenal cell membrane depolarization or increasing intracellular Ca^2+^ concentration, leading to aldosterone synthase (CYP11B2) overexpression and subsequent increasing aldosterone production. The prevalence of APAs with somatic mutations increases up to 90% when genotyping is performed using CYP11B2 immunohistochemistry-guided next generation sequencing techniques (13). *KCNJ5* is the most prevalent somatic mutation gene in East Asia, European and American populations, with the prevalence varied from 70% to 43% (14). While in black population, the prevalence of *KCNJ5* mutation is second to *CACNA1D* alteration, the latter is 42% (13).

Apart from somatic mutations in APA, genetic alterations have also been detected in patients with familial form of hyperaldosteronism. To date, four forms of familial aldosteronism (FH I, FHII, FH III and FH IV) have been reported. The FH-I or glucocorticoid remediable aldosteronism (GRA) was caused by a chimeric *CYP11B1/CYP11B2* gene, with which the aldosterone overproduction was regulated by ACTH (15). FH II, FH III and FH IV was caused by germline mutation in *CLCN2*, *KCNJ5* and *CACNA1H* gene, respectively (5, 9, 10).

We herein present a case of a young female patient who underwent complete left adrenalectomy due to APA 10 years ago. While her PA resolved following the treatment, it recently recurred due to an APA on the right adrenal gland.

## Case Report

**Figure 1:**
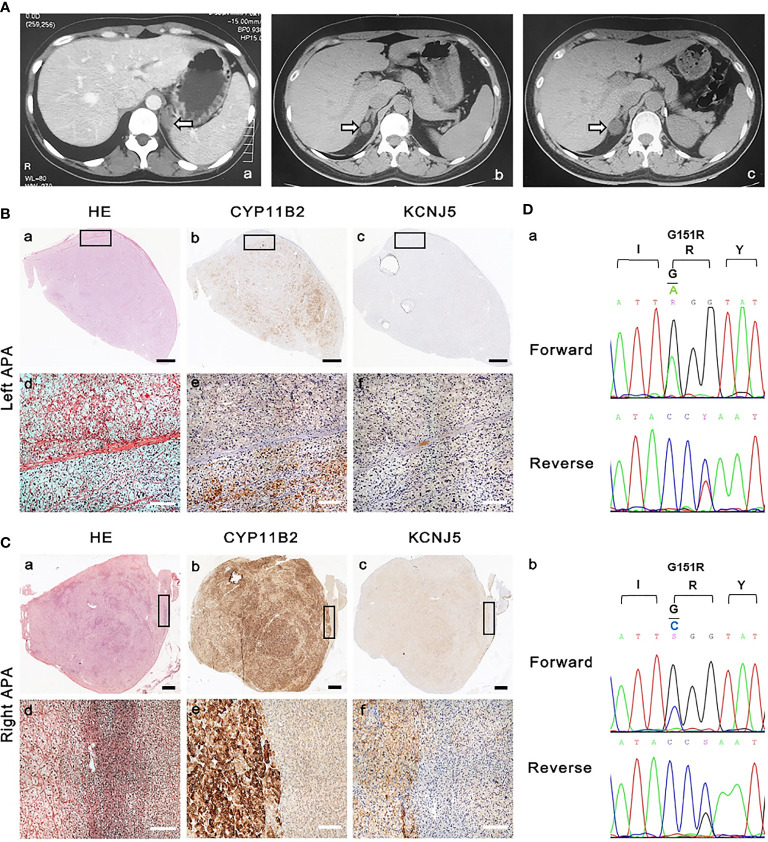
Imaging, histology, and genetic studies of left and right aldosterone-producing-adenomas. **(A)** Adrenal computed tomography scans according to year of acquisition: a. 2004; b. 2014; c. 2017. The arrows indicate the adrenal adenoma with the largest diameter. Hematoxylin–eosin (HE) staining and immunohistochemistry (see Document, **Supplemental Digital Content 1**, which describes detailed methods) of **(B)** left and **(C)** right aldosterone-producing adenomas: a, d. HE staining; b, e. CYP11B2 immunohistochemical staining of aldosterone synthase; c, f. immunohistochemical staining of *KCNJ5*. Black scale bar=2mm; white scale bar=100μm. CYP11B2, aldosterone synthase; KCNJ5, G-protein–coupled inwardly rectifying potassium channel. **(D)** Sanger DNA sequences of mutations in *KCNJ5* gene and corresponding amino-acid change (see Document, **Supplemental Digital Content 1**): a. Left APA: c. 451G>A, p.G151R; b. Right APA: c. 451G>C, p.G151R.

A 29-year-old female patient was admitted to our hospital in 2004. She reportedly had hypertension for one year. Her father had hypertension. Her systolic and diastolic blood pressures (BP) ranged from 150 to 190 and 110 to 120 mmHg, respectively, and she was treated with sustained-release felodipine (5 mg once a day [QD]). On admission, the physical examination showed her office blood pressure was 180/100mmHg. Her body mass index was 22.6 kg/m^2^. She was found to have hypokalemia, high plasma aldosterone levels, and suppressed PRA (**Table 1**). An adrenal computed tomography (CT) scan showed a left 2.0 × 1.5-cm nodule (**Figure 1A**, image a). She was diagnosed with PA after exclusion of other secondary forms of hypertension, including pheochromocytoma, Cushing syndrome, and renal artery stenosis (data not shown), and underwent laparoscopic left adrenalectomy. The resected adrenal gland contained a 2.0 × 2.0-cm APA, which was immediately confirmed by hematoxylin and eosin (HE) staining (**Figure 1B**, slide a) and again in 2020 by CYP11B2 staining (**Figure 1B**, slide b). The postoperative course was uneventful, the hypokalemia was corrected immediately after the surgery, and her blood pressure remained normal without medication (**Table 1**) for the following 10 years.

**Table 1:** Baseline and post-left-adrenalectomy clinical characteristics in 2004.

	Before adrenalectomy	After adrenalectomy
**SBP (mmHg)**	180	120
**DBP (mmHg)**	100	90
**Serum potassium (mmol/L)**	2.39	4.00
**Plasma aldosterone (pg/ml)**	469.8	/
**Plasma renin activity (ng/ml/h)**	0.1	/
**Serum creatinine (μmol/L)**	50	/
**Antihypertensive medication (n)**	1	0

DBP, Diastolic blood pressure; SBP, Systolic blood pressure./: value not obtained.

In March 2014, a routine physical examination showed that her blood pressure had elevated to 170/100 mmHg. Outpatient examination showed normokalemia (4.12 mmol/L) and no suppression of PRA (2.56 ng/ml/h), while the plasma aldosterone concentration (PAC) was elevated to 371.9 pg/ml. She was treated with 90 mg sustained-release diltiazem twice a day (BID), and her blood pressure returned to normal. One month later, her aldosterone, PRA, and serum potassium levels were re-evaluated. Mild hypokalemia (3.46 mmol/L) was detected with PAC and PRA levels of 339.83 pg/ml and 1.29 ng/ml/h, respectively. The patient was admitted for PA screening. A supine saline infusion test was performed, and the results were positive with post-test PAC level recorded at 222.86 pg/ml. Recurrence of PA was thus confirmed. Meanwhile, the adrenal CT scan revealed a 1.6 × 1.6-cm nodule on the right adrenal gland, which had not been present in 2004 (**Figure 1A**, image b). Other forms of secondary hypertension were excluded. She was administered 20 mg of spironolactone and 5 mg of amlodipine QD and was monitored regularly; her BP was maintained at around 110-130/80-90 mmHg, and her serum potassium levels at around 4 mmol/L. Her father was screened for PA in this period and the diagnosis was primary hypertension.

From October 2016, her BP rose gradually, and the antihypertensive regimen was adjusted accordingly. In May 2017, she was treated with candesartan (8 mg QD), carvedilol (10 mg BID), amlodipine (5 mg QD), and spironolactone (20 mg BID). Her BP was maintained at approximately 150-160/100-110 mmHg, and her serum potassium levels at around 3.9 mmol/L. A repeat adrenal CT scan showed an enlarged right adrenal nodule that measured 2.5 × 1.8 cm. In 2018, her BP became difficult to control (approximately 170/100 mmHg), and she was admitted to the hospital for further evaluation. She was treated with sustained-release verapamil (360 mg QD), terazosin (2 mg every night), amlodipine (5 mg QD), and potassium chloride sustained-release tablets (2 g three times a day) for 4 weeks before admission. As shown in **Table 2**, her PRA was suppressed with high plasma and urinary aldosterone levels. She underwent a saline infusion test, and the post-test PAC level was recorded at 514.56 pg/ml. The diagnosis of PA was confirmed, and other secondary forms of hypertension were excluded. She underwent partial laparoscopic right adrenalectomy to remove the adenoma. The resected adrenal gland measured 2.5 × 2.5 × 2.0 cm and was confirmed by HE and CYP11B2 staining to contain a 1.5 × 1.5 × 0.3 cm APA (**Figure 1C**, slides a and b).

**Table 2:** Baseline and post-right-adrenalectomy clinical characteristics.

	Before right adrenalectomy (2014)	Before right adrenalectomy (2018)	Post-adrenalectomy (1 month)	Post-adrenalectomy (12 months)	Post-adrenalectomy (36 months)
**SBP (mmHg)**	138	133	130	140	120
**DBP (mmHg)**	86	72	85	92	80
**24h SBP (mmHg)**	129	137	139	/	122
**24h DBP (mmHg)**	89	92	92	/	78
**Serum potassium (mmol/L)**	3.46	3.59*	4.34	3.92	4.0
**Plasma aldosterone (pg/ml)**	339.83	1037.96	91.53	92.43	155.17
**Plasma renin activity (ng/ml/h)**	1.29	0.66	6.96	1.02	2.55
**Urinary aldosterone (μg/24h)**	25.38	37.2	2.86	/	/
**Urinary free cortisol (μg/24h)**	148.56	109.08	33.12	/	64.64
**ACTH (pg/ml)**	/	20.34	77.41	/	65.50
**Creatinine (μmol/L)**	53	58	76	63	75
**Antihypertensive medication (n)**	2	4	0	1	2

ACTH, adrenocorticotropic hormone; DBP, diastolic blood pressure; SBP, systolic blood pressure; *without potassium chloride supplementation;/: value not obtained.

One month after surgery, her serum potassium levels increased to 4.34 mmol/L without potassium supplementation, and the PAC and PRA returned to normal levels (**Table 2**). Subclinical hypocortisolism was observed. Her office BP was normal, but ambulatory BP was still elevated. She was then administered candesartan (8 mg QD) and carvedilol (10 mg QD), and her BP was maintained at approximately 120/80 mmHg. **Figure S1** illustrated a timeline with relevant data retracing the different steps of diagnosis and treatment of primary aldosteronism from 2004 to 2021. The patient will be followed regularly every six months with blood pressure measurement and antihypertensive medication adjustment if necessary. Biochemical measurement including plasma aldosterone, renin activity and potassium are tested annually.

To explore the genetics of APA, DNA Sanger sequencing was performed on the slices of the left and right adenomas. The results showed that both adenomas carried mutations in the *KCNJ5* gene G151R with different base changes (Left: c.451G > A; Right c.451G > C) (**Figure 1D**, graphs a and b). The mutations were confirmed by extracting DNA from the remaining slices and subsequent polymerase chain reaction and Sanger sequencing. The immunostaining of *KCNJ5* were decreased in the CYP11B2-positive stained adenomas (**Figure 1B** slide c; **Figure 1C**, slide c). Whole exome sequencing was conducted on 21,522 genes. A panel of 140 genes (**Table S1**) associated with blood pressure and electrolyte dysregulation, mainly related to adrenal gland diseases, renal diseases and metabolic disorders, were further analyzed in depth. 49 genes of them were detected to carry variants (**Table S2**), including those known to be related to familial aldosteronism and aldosterone-producing adenoma. We found three variants, CACNA1H c.1919C>T, CACNA1H c.6212G>A, and KCNJ5 c.844C>G. Since all of them have an allele frequency >5% in gnomAD database (http://gnomad.broadinstitute.org/), and because all have a neutral effect on the change of the amino acid sequence predicted by the in-silico prediction tools (Mutation Taster, PROVEAN and SIFT), and none of them was recorded by the disease databases (OMIM, Clinvar), these three variants are classified as benign according to the 2015 ACMG classification (16). We also performed long-range polymerase chain reaction to detect the chimeric CYP11B1/B2 gene, and the result was negative.

## Discussion

The present report documents the case of a young female patient with recurrent PA after left adrenalectomy due to an APA that was treated 10 years previously.

This patient was first diagnosed with PA in 2004, when she was 29 years old. At that time, her diagnosis was based on the following symptoms: hypertension, hypokalemia, high PAC, and left adrenal adenoma, but no confirmatory tests. However, according to the 2016 Endocrine Society Primary Aldosteronism Management Guideline (17), the diagnosis was valid because she had a florid PA phenotype with hypokalemia and a PAC higher than 200 ng/dl. She did not undergo adrenal venous sampling (AVS) to identify the dominant side in 2004, as our department was not equipped to perform the test at that time. However, the patient fulfilled the criteria for skipping AVS according to current guidelines: PAC >300 ng/dl, unilateral adenoma > 1 cm in size and clear on the contralateral side, age < 35 years (18). Whether defined by clinical or biochemical measures, the current PASO (Primary Aldosteronism Surgical Outcome) study consensus seemed to indicate the complete remission of PA after the first left adrenalectomy (2).

The recurrence of PA 10 years later due to contralateral APA is interesting and requires further exploration. This patient’s diagnosis of recurrent PA was validated by a repeat saline infusion confirmatory test after withdrawing interfering antihypertensive medications. She belongs to the sporadic PA form according to the result of exome sequencing on germline DNA. Although 49/140 genes which are related to blood pressure and electrolyte dysregulation were detected to carry nucleotide variations, none of them were pathogenic in accordance with ACMG classification (16). To the best of our knowledge, few reports have considered the recurrence of PA after complete unilateral adrenalectomy (3, 4). Citton et al. reported that 3.7% (3/81 cases) of patients with biochemically resolved PA experienced recurrence after a mean follow-up of 64 months (4). Of these three patients, two were female, two had a single APA, and one had multinodular adrenal hyperplasia. A separate pathological study of the removed adrenal gland revealed the existence of several micronodules surrounding the largest APA, which could not be identified by the adrenal CT scan at an early stage (19). These micronodules might be functional and subsequently develop into apparent APAs (20). Therefore, recent guidelines highly recommend the complete removal of the adrenal gland over its partial resection (17, 21). These reports inform our speculation that the “normal” right adrenal gland visualized on the CT image obtained in 2004 contained a micronodule that gradually developed into apparent APA. Another reason for the recurrence of PA 10 years after the left adrenalectomy with a newly developed APA on the contralateral adrenal side might be related to the somatic mutation in the aldosterone-driver gene *KCNJ5* (5), which was identified on both left and right APAs. We previously reported that the prevalence of *KCNJ5* mutations in APA in Chinese patients was as high as 76.8% (22). In our case, the two adenomas carried the same *KCNJ5* mutation, G151R, but with different nucleotide changes. However, the underlying mechanism is unclear. Nanba et al. reported that multiple APAs of a single adrenal gland harbored independent somatic mutations and that different mutations could coexist in a single APA (13). Further, the aldosterone-producing microadenoma (APM, also previously called aldosterone-producing cell cluster), which is considered a precursor of APA and part of the normal adrenal gland, can produce aldosterone constitutively and might develop into APA when harboring the *KCNJ5* gene mutation (23). However, the mechanism governing the development of APA on both adrenal sides within 10 years remains unknown. Further research and more case reports are required to better elucidate the pathogenetic mechanism. In the clinic, patients are usually followed more frequently in the period immediately after adrenalectomy, but not in the long term (24). Furthermore, when patients were considered to have been “cured” based on the maintenance of BP without medication and by the correction of hypokalemia, the recurrence of hypertension is usually considered to be the onset of existing primary hypertension prompted by aging or others risk factors. However, the present and other reports concerning APM remind us that the recurrence of PA after adrenalectomy may be an issue throughout life. Untreated excess aldosterone production will eventually cause cardiovascular damage or death, requiring patients with PA who underwent surgery to be followed up in the long term. This strategy is in accordance with the recent consensus and guidelines on the management of patients with PA (21, 25, 26). We further suggest that, in addition to serum potassium levels, aldosterone and renin concentrations be rechecked annually and that adrenal gland CT be administered if necessary.

## Conclusion

We herein report the rare case of a young female patient who presented with recurred PA and a right APA after undergoing adrenalectomy due to a left APA 10 years ago. The two adrenal glands carried the same aldosterone-driver *KCNJ5* gene mutation, but with different nucleotide changes. We suggest that patients who undergo adrenalectomy for APA should be followed up for life to prevent the detrimental cardiovascular effects caused by the recurrence of PA.

## Data Availability Statement

The datasets presented in this study can be found in online repositories. The names of the repository/repositories and accession number(s) can be found in the article/**Supplementary Material**.

## Ethics Statement

The studies involving human participants were reviewed and approved by Ruijin Hospital Ethics Committee Shanghai Jiao Tong University School of Medicine. The patients/participants provided their written informed consent to participate in this study.

## Author Contributions

LK and JZ collected the clinical data, wrote the manuscript. LD analyzed and interpreted the pathological and the molecular genetic data. JX participated the follow-up of the patient. P-JG and J-GW contributed to writing the manuscript. All authors contributed to the article and approved the submitted version.

## Funding

This work was supported by a research grant by Shanghai Bureau of Health and Family Planning (Grant number 201740079 to LZ).

## Conflict of Interest

The authors declare that the research was conducted in the absence of any commercial or financial relationships that could be construed as a potential conflict of interest.

## Publisher’s Note

All claims expressed in this article are solely those of the authors and do not necessarily represent those of their affiliated organizations, or those of the publisher, the editors and the reviewers. Any product that may be evaluated in this article, or claim that may be made by its manufacturer, is not guaranteed or endorsed by the publisher.
