# Supplementary material for: Recurrence of Primary Aldosteronism 10 Years After Left Adrenalectomy for Aldosterone-Producing Adenoma: A Case Report
Source: Front Endocrinol (Lausanne). 2021 Sep 24;12:728595. doi: 10.3389/fendo.2021.728595 (PMC8498213; doi:10.3389/fendo.2021.728595)
Supplement: Supplementary file 1 [file DataSheet_1.docx]

**Supplemental Digital Content 1**

**Methods**

*Blood pressure measurement*

1. Office blood pressure measurement

In 2004, the blood pressure measurement in our hospital was carried out using a standard mercury sphygmomanometer. Since 2014, BP was measured with the 7051 automated BP monitor (Omron Healthcare). Two consecutive blood pressure readings measured with 1-minute interval were averaged and recorded.

1. Ambulatory blood pressure measurement

Oscillometric SpaceLabs 90207 monitors (SpaceLabs Inc) was used to obtain 24-hour ambulatory blood pressure readings at 20-minute intervals.

*Laboratory assays*

All tests were done in a College of American Pathologists (number 7217913)-accredited laboratory. Plasma aldosterone and plasma renin activity were measured by RIA following manufacturer’s instructions (A Beckman Coulter Corp). The intraassay and interassay coefficients of variation were 9.3% and 9.5% for aldosterone and 10.1% and 10.2% for renin activity, respectively. The normal value is 3.81–31.33 ng/dL and 0.1–5.5 ng/mL^.^h, respectively. Serum cortisol and serum ACTH were measured by immunoluminescence and RIA following the manufacturer’s instructions (A Beckman Coulter Corp). The intraassay and interassay coefficients of variation were 6.7% and 7.9% for cortisol and 6.1% and 5.3% for ACTH, respectively. The normal value is 6.7–22.6 g/dL and 12–78 pg/mL, respectively (1).

*Immunohistochemistry*

Adrenal specimens were fixed in 10% formalin and embedded in paraffin after surgery. Selected blocks were serially sectioned and stained with hematoxylin and eosin (HE) and cytochrome P450 family 11 subfamily B member 2 (*CYP11B2)* (Merck Millipore, MABS1251; 1:200 dilution), as well as potassium inwardly rectifying channel subfamily J member (*KCNJ5)* antibody (SIGMA, HPA017353; 1:200 dilution), as previously described (2, 3).

*Sanger sequencing*

Following the lysis of the 35-mm tumor slices, nucleic acids were obtained using Qiagen column separation according to the manufacturer’s instructions (Qiagen, Hilden, Germany). *KCNJ5* mutations located in exon 2 were analyzed by direct DNA sequencing. Genomic DNA was amplified by polymerase chain reaction (PCR) (forward primer: GGTGACCTGGACCATGTTGGCG; reverse primer: CTTGGCAGGTCATGCCTGTGGC5). All PCR amplimers were checked by 1% agarose gel electrophoresis. Sequencing reactions were performed using a BigDye Terminator Cycle Sequencing Kit (Thermo Fisher, Waltham, Massachusettsm, USA) and analyzed on a 24-capillary 3500 DX DNA Analyzer (Applied Biosystems, USA). Exonic sequences were read and aligned using Chromas software (Technelysium, Version 1.62).

*Whole exome sequencing*

Whole exome sequencing was performed at BestNovo Diagnostics Lab (Beijing, China). Briefly, genomic DNA was extracted from peripheral blood leukocytes (QIAGEN, Hilden, Germany). Exome capture was prepared based on the protocols of Agilent SureSelect QXT Target Enrichment for Illumina Multiplexed Sequencing version E0. The libraries were prepared based on the protocols of Agilent SureSelect QXT Library Prep Kit (5500-0127). Illumina NovaSeq 6000 S2 Reagent Kit (300 cycles) was used for paired-end 2×150 bp sequencing on an Illumina NovaSeq 6000 System.

# References

1. Jiang Y, Zhang C, Wang W, Su T, Zhou W, Jiang L, et al. Diagnostic value of ACTH stimulation test in determining the subtypes of primary aldosteronism. *J Clin Endocrinol Metab* (2015) 100(5):1837-44. Epub 2015/02/20. doi: 10.1210/jc.2014-3551. PubMed PMID: 25695882.

2. Omata K, Anand SK, Hovelson DH, Liu CJ, Yamazaki Y, Nakamura Y, et al. Aldosterone-Producing Cell Clusters Frequently Harbor Somatic Mutations and Accumulate With Age in Normal Adrenals. *J Endocr Soc* (2017) 1(7):787-99. Epub 2017/12/22. doi: 10.1210/js.2017-00134. PubMed PMID: 29264530; PubMed Central PMCID: PMCPMC5686701.

3. Zhou J, Shaikh LH, Neogi SG, McFarlane I, Zhao W, Figg N, et al. DACH1, a zona glomerulosa selective gene in the human adrenal, activates transforming growth factor-beta signaling and suppresses aldosterone secretion. *Hypertension* (2015) 65(5):1103-10. Epub 2015/03/18. doi: 10.1161/HYP.0000000000000025. PubMed PMID: 25776071; PubMed Central PMCID: PMCPMC4387203.
