# Supplementary figures and images for: Recurrence of Primary Aldosteronism 10 Years After Left Adrenalectomy for Aldosterone-Producing Adenoma: A Case Report
Source: Front Endocrinol (Lausanne). 2021 Sep 24;12:728595. doi: 10.3389/fendo.2021.728595 (PMC8498213; doi:10.3389/fendo.2021.728595)

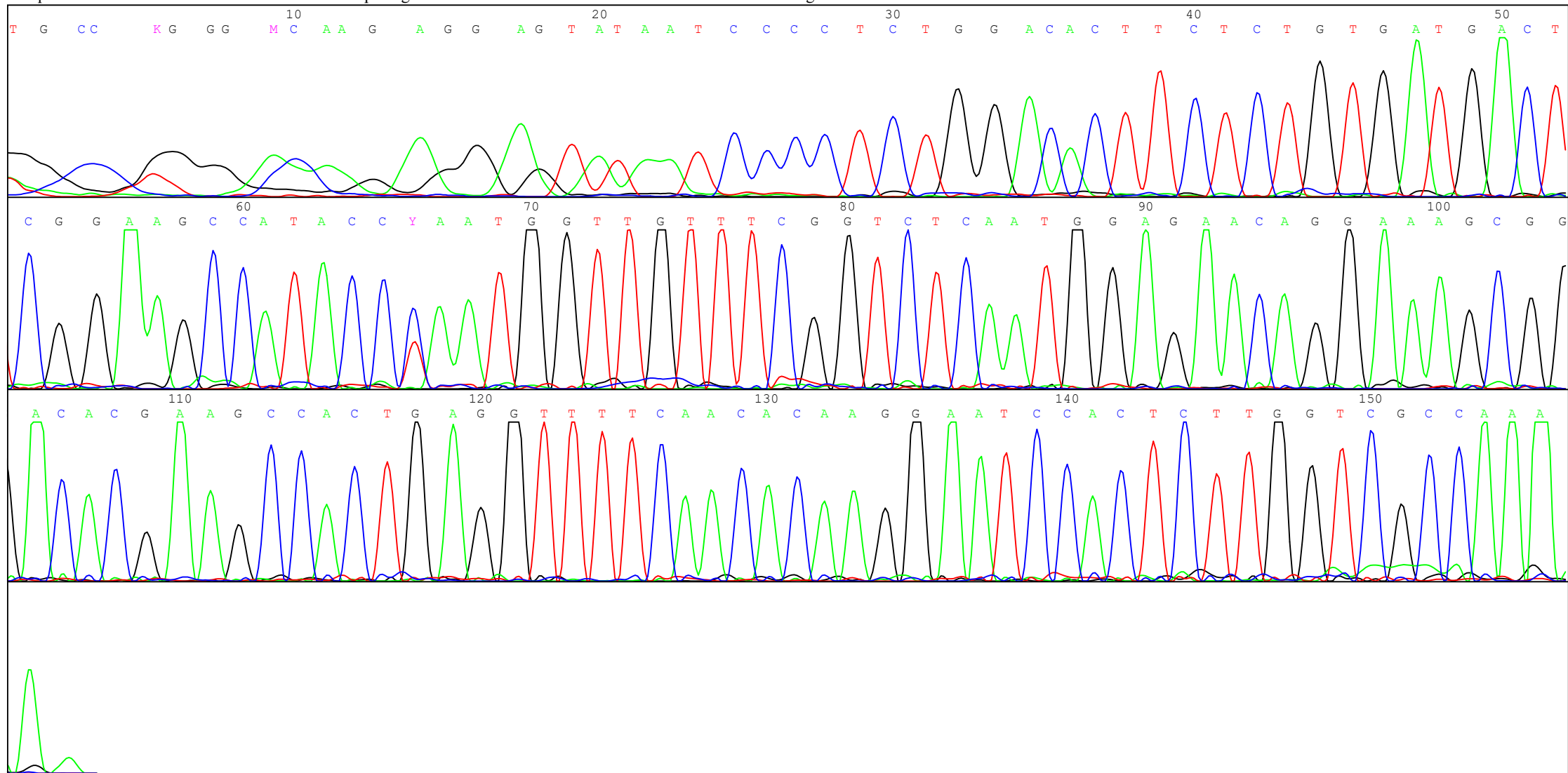

Supplement: Supplementary file 3 [file DataSheet_3.pdf]

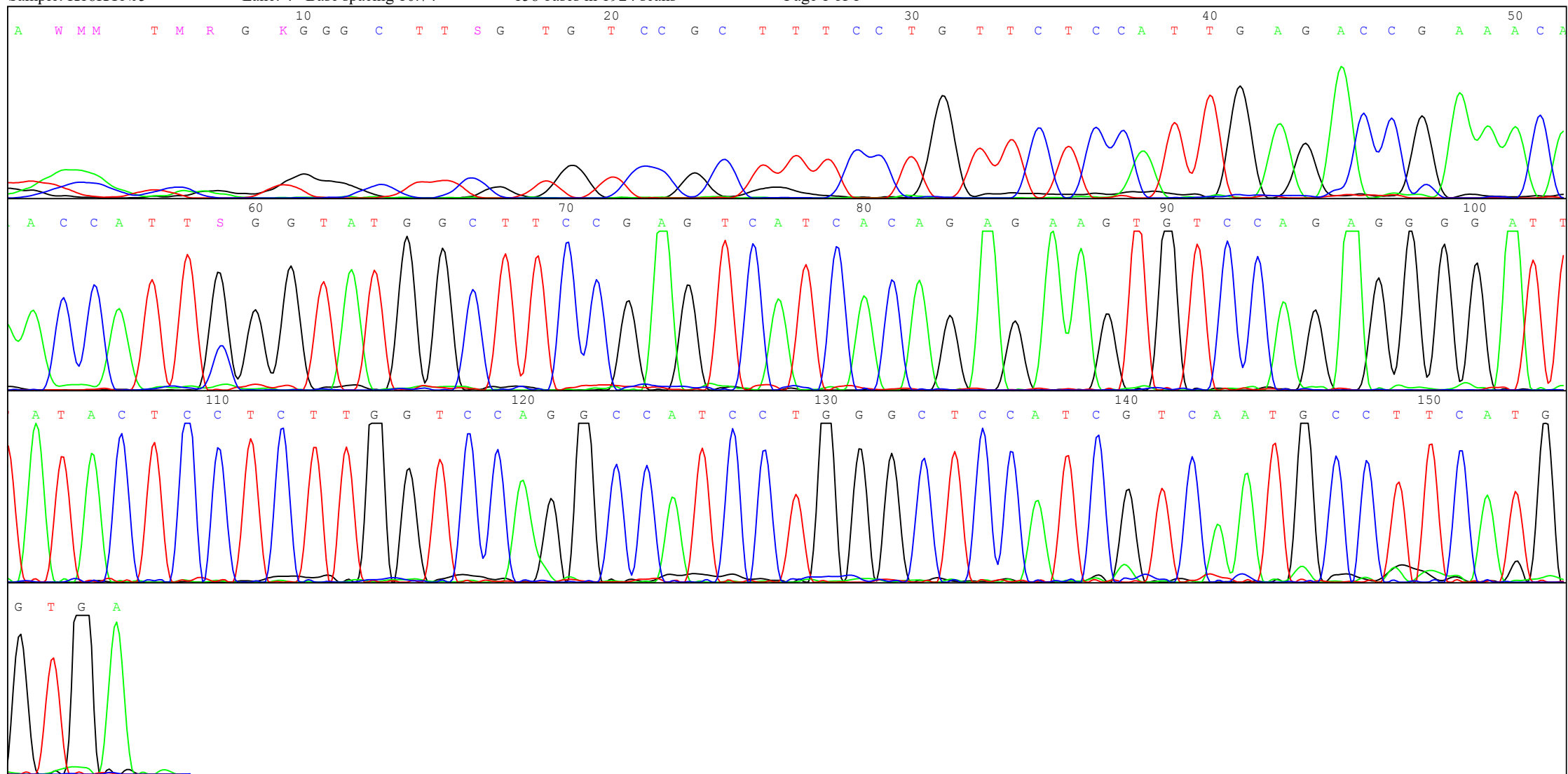

Supplement: Supplementary file 4 [file DataSheet_4.pdf]

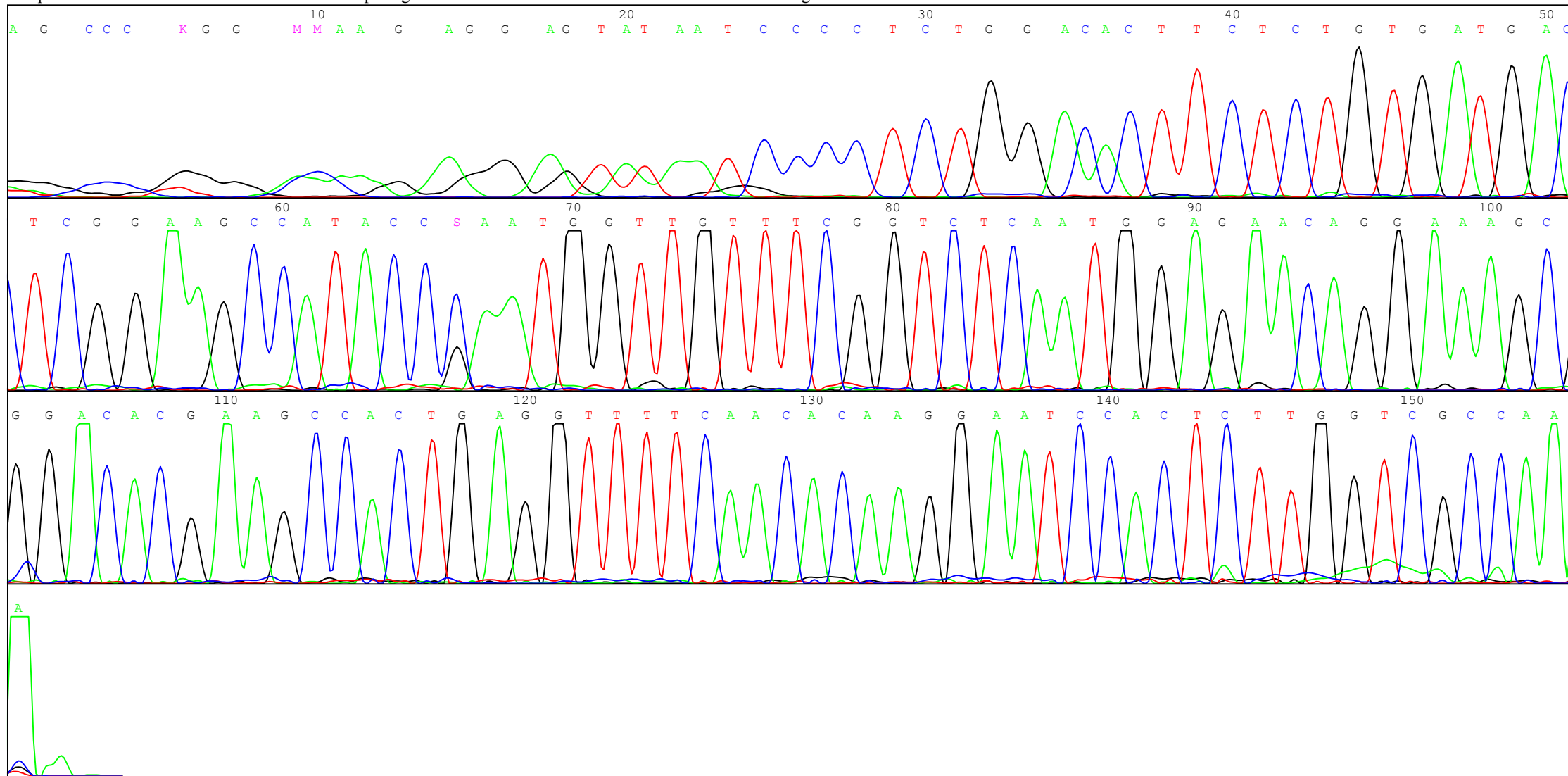

Supplement: Supplementary file 5 [file DataSheet_5.pdf]

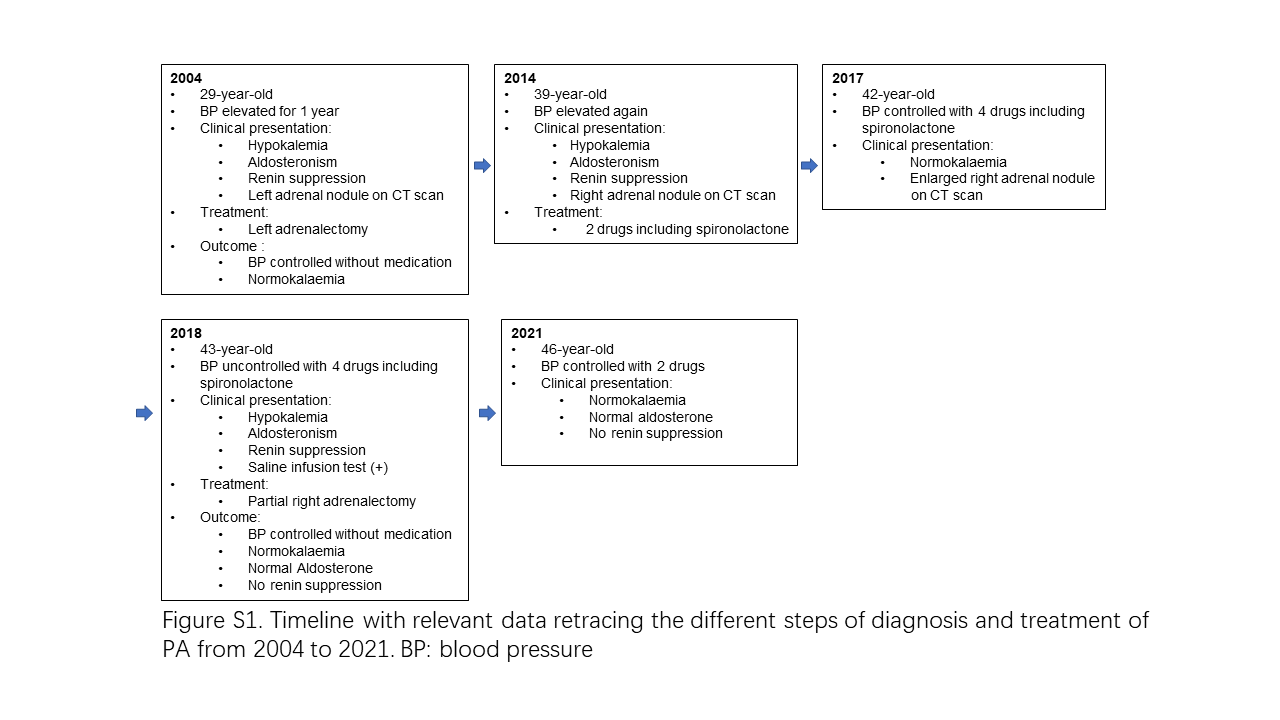

Supplement: Supplementary file 6 [file Image_1.tif]
