# Supplementary material for: Recurrence of Primary Aldosteronism 10 Years After Left Adrenalectomy for Aldosterone-Producing Adenoma: A Case Report
Source: Front Endocrinol (Lausanne). 2021 Sep 24;12:728595. doi: 10.3389/fendo.2021.728595 (PMC8498213; doi:10.3389/fendo.2021.728595)
Supplement: Supplementary file 7 [file Table_1.docx]

**Table S1. List of 140 genes in the deep analysis**

| Gene name |
| --- |
| ABCC6, ACE, ADA2, ADD1, ADRB2, ADRB3, AGRP, AGT, AGTR1, ALMS1, ARMC5, ATP1A1, ATP1B1, ATP2B3, ATRX, AVPR2, BSND, CACNA1D, CACNA1H, CARTPT, CASR, CCND1, CECR1, CEP19, CFH, CFHR1, CFHR3, CLCN2, CLCNKA, CLCNKB, COL4A3, COL4A4, COL4A5, CPOX, CUL3, CTNNB1, CYP11A1, CYP11B1, CYP17A1, CYP21A2, CYP3A5, DNAJB11, DYRK1B, DZIP1L, ECE1, EGFR, EGLN1, ENPP1, EPAS1, ERCC6, ERCC8, FGFR1, FH, FMO3, FPLD1, FN1, G6PC, GUCY1A3, GANAB, GBE1, GDNF, GHRL, GNB3, GUCY1A1, HMBS, HRAS, HSD11B2, HSD17B1, HSD3B2, KCNJ1, KCNJ5, KCNMB1, KIF1B, KLHL3, LEP, LMNA, LEPR, MAX, MC4R, MEN1, MET, MTTP, MUC1, NF1, NF2, NME1, NOS2, NOS3, NOTCH2, NR3C1, NR3C2, PDE11A, PDE3A, PDE8B, PKD1, PKD2, PKHD1, PLIN1, POMC, PPARG, PPARGC1B, PRKACA, PRKAR1A, PRKCSH, PTGIS, REN, RET, RGS5, SCN2B, SCNN1A, SCNN1B, SCNN1G, SDC3, SDHA, SDHAF2, SDHB, SDHC, SDHD, SEC63, SERPINA6, SIM1, SLC12A1, SLC12A3, SLC2A10, SLC37A4, SMAD4, SMARCAL1, STAR, SUGCT, TMEM127, TP53, UCP1, UCP3, VHL, WNK1, WNK4, WT1, XYLT1, XYLT2, YY1AP1 |

**NOTE:** Most of these genes have been reported in the following diseases: familial hyperaldosteronism, aldosterone-producing adenomas, primary pigmented nodular adrenocortical disease, pheochromocytoma-paraganglioma, apparent mineralocorticoid excess, hypertension exacerbation in pregnancy, Bartter syndrome, Gitelman syndrome, Liddle syndrome, Gordon syndrome, polycystic kidney, morbid obesity, hypertension and brachydactyly Syndrome, etc..
