# Supplementary material for: Recurrence of Primary Aldosteronism 10 Years After Left Adrenalectomy for Aldosterone-Producing Adenoma: A Case Report
Source: Front Endocrinol (Lausanne). 2021 Sep 24;12:728595. doi: 10.3389/fendo.2021.728595 (PMC8498213; doi:10.3389/fendo.2021.728595)
Supplement: Supplementary file 8 [file Table_2.docx]

| **Gene** | **Transcript** | **Exon** | **Bases Variant** | **Amino-acid Variant** | **Status** | **Pathogenecity** |
| --- | --- | --- | --- | --- | --- | --- |
| CFH | NM_000186 | exon4 | c.418A>G | p.Ile140Val | HET | Benign |
| EPAS1 | NM_001430 | exon6 | c.574-5C>G | - | HET | Benign |
| ALMS1 | NM_015120 | exon8 | c.1930C>G | p.Pro644Ala | HET | Benign |
| DZIP1L | NM_173543 | exon13 | c.1739A>G | p.His580Arg | HET | Benign |
| PKHD1 | NM_138694 | exon32 | c.4201G>T | p.Ala1401Ser | HET | Benign |
| SUGCT | NM_001193311 | exon6 | c.499A>C | p.Ile167Leu | HET | Benign |
| CYP3A5 | NM_000777 | exon7 | c.599A>G | p.Gln200Arg | HET | Benign |
| SLC12A1 | NM_000338 | exon12 | c.1536T>C | p.Leu512Leu | HET | Benign |
| KIF1B | NM_015074 | exon29 | c.3260A>G | p.Tyr1087Cys | HET | Benign |
| CLCNKA | NM_001257139 | exon9 | c.815A>T | p.Tyr272Phe | HET | Benign |
| CLCNKA | NM_001257139 | exon13 | c.1210G>A | p.Ala404Thr | HET | Benign |
| CLCNKB | NM_000085 | exon2 | c.80G>T | p.Arg27Leu | HET | Benign |
| CLCNKB | NM_000085 | exon7 | c.641C>G | p.Ala214Gly | HOM | Benign |
| CLCNKB | NM_001165945 | exon2 | c.353C>T | p.Ala118Val | HOM | Benign |
| CLCNKB | NM_001165945 | exon9 | c.1178T>C | p.Met393Thr | HOM | Benign |
| SDC3 | NM_014654 | exon4 | c.986C>T | p.Thr329Ile | HOM | Benign |
| LEPR | NM_001198687 | exon3 | c.326A>G | p.Lys109Arg | HOM | Benign |
| LEPR | NM_001198687 | exon5 | c.668A>G | p.Gln223Arg | HOM | Benign |
| NOTCH2 | NM_001200001 | exon2 | c.112G>A | p.Glu38Lys | HET | Benign |
| NOTCH2 | NM_001200001 | exon1 | c.61G>A | p.Ala21Thr | HET | Benign |
| NOTCH2 | NM_001200001 | exon1 | c.57C>G | p.Cys19Trp | HET | Benign |
| CFH | NM_000186 | exon9 | c.1204C>T | p.His402Tyr | HOM | Benign |
| CFH | NM_000186 | exon18 | c.2808G>T | p.Glu936Asp | HOM | Benign |
| AGT | NM_000029 | exon2 | c.803T>C | p.Met268Thr | HET | Benign |
| EGLN1 | NM_022051 | exon1 | c.380G>C | p.Cys127Ser | HOM | Benign |
| ALMS1 | NM_015120 | exon8 | c.2012T>G | p.Val671Gly | HOM | Benign |

**Table S2. Variants detected in 140 genes with deep analysis**

| ALMS1 | NM_015120 | exon19 | c.12086G>A | p.Arg4029Lys | HOM | Benign |
| --- | --- | --- | --- | --- | --- | --- |
| PDE11A | NM_001077196 | exon5 | c.245-3T>C | - | HET | Benign |
| PDE11A | NM_016953 | exon1 | c.604C>T | p.Arg202Cys | HET | Benign |
| PDE11A | NM_016953 | exon1 | c.551G>A | p.Arg184Gln | HOM | Benign |
| FN1 | NM_212474 | exon39 | c.5878G>A | p.Val1960Ile | HOM | Benign |
| FN1 | NM_001306129 | exon17 | c.2449A>C | p.Thr817Pro | HOM | Benign |
| FN1 | NM_001306131 | exon2 | c.149-3C>T | - | HOM | Benign |
| FN1 | NM_001306129 | exon1 | c.44A>T | p.Gln15Leu | HOM | Benign |
| CUL3 | NM_001257197 | exon11 | c.1501G>A | p.Val501Ile | HOM | Benign |
| COL4A4 | NM_000092 | exon44 | c.4207T>C | p.Ser1403Pro | HET | Benign |
| COL4A4 | NM_000092 | exon42 | c.3979G>A | p.Val1327Met | HET | Benign |
| COL4A4 | NM_000092 | exon33 | c.3011C>T | p.Pro1004Leu | HET | Benign |
| COL4A4 | NM_000092 | exon21 | c.1444C>T | p.Pro482Ser | HET | Benign |
| COL4A3 | NM_000091 | exon7 | c.422T>C | p.Leu141Pro | HOM | Benign |
| COL4A3 | NM_000091 | exon9 | c.485A>G | p.Glu162Gly | HOM | Benign |
| COL4A3 | NM_000091 | exon21 | c.1223G>A | p.Arg408His | HET | Benign |
| COL4A3 | NM_000091 | exon22 | c.1352A>G | p.His451Arg | HET | Benign |
| COL4A3 | NM_000091 | exon25 | c.1721C>T | p.Pro574Leu | HET | Benign |
| GHRL | NM_001134944 | exon2 | c.178C>A | p.Leu60Met | HET | Benign |
| GBE1 | NM_000158 | exon8 | c.1000A>G | p.Ile334Val | HOM | Benign |
| GBE1 | NM_000158 | exon5 | c.568A>G | p.Arg190Gly | HOM | Benign |
| CPOX | NM_000097 | exon4 | c.814A>C | p.Asn272His | HET | Benign |
| CASR | NM_000388 | exon7 | c.3031G>C | p.Glu1011Gln | HOM | Benign |
| DZIP1L | NM_173543 | exon14 | c.1933A>G | p.Lys645Glu | HET | Benign |
| DZIP1L | NM_173543 | exon13 | c.1778G>A | p.Arg593His | HET | Benign |
| DZIP1L | NM_173543 | exon13 | c.1633A>G | p.Thr545Ala | HET | Benign |
| DZIP1L | NM_173543 | exon6 | c.961C>T | p.Arg321Trp | HET | Benign |
| CEP19 | NM_032898 | exon2 | c.7A>G | p.Met3Val | HOM | Benign |
| ADD1 | NM_001119 | exon10 | c.1378G>T | p.Gly460Trp | HOM | Benign |
| ADD1 | NM_001119 | exon13 | c.1757C>G | p.Ser586Cys | HOM | Benign |

| MTTP | NM_001300785 | exon7 | c.972C>G | p.His324Gln | HET | Benign |
| --- | --- | --- | --- | --- | --- | --- |
| UCP1 | NM_021833 | exon5 | c.685A>T | p.Met229Leu | HET | Benign |
| UCP1 | NM_021833 | exon2 | c.190G>A | p.Ala64Thr | HET | Benign |
| NR3C2 | NM_000901 | exon2 | c.1661A>G | p.Asn554Ser | HET | Benign |
| NR3C2 | NM_000901 | exon2 | c.538G>A | p.Val180Ile | HOM | Benign |
| ADRB2 | NM_000024 | exon1 | c.46G>A | p.Gly16Arg | HET | Benign |
| ADRB2 | NM_000024 | exon1 | c.79G>C | p.Glu27Gln | HOM | Benign |
| PPARGC1B | NM_001172698 | exon4 | c.677G>A | p.Arg226Gln | HET | Benign |
| PKHD1 | NM_138694 | exon67 | c.12143A>G | p.Gln4048Arg | HET | Benign |
| PKHD1 | NM_138694 | exon66 | c.11696A>G | p.Gln3899Arg | HET | Benign |
| PKHD1 | NM_138694 | exon35 | c.5608T>G | p.Leu1870Val | HOM | Benign |
| PKHD1 | NM_138694 | exon32 | c.3785C>T | p.Ala1262Val | HET | Benign |
| PKHD1 | NM_138694 | exon24 | c.2489A>G | p.Asn830Ser | HET | Benign |
| PKHD1 | NM_138694 | exon22 | c.2278C>T | p.Arg760Cys | HET | Benign |
| SIM1 | NM_005068 | exon9 | c.1112C>T | p.Ala371Val | HET | Benign |
| SIM1 | NM_005068 | exon9 | c.1054C>A | p.Pro352Thr | HET | Benign |
| MET | NM_000245 | exon2 | c.1124A>G | p.Asn375Ser | HET | Benign |
| NOS3 | NM_001160109 | exon7 | c.894T>G | p.Asp298Glu | HOM | Benign |
| ERCC6 | NM_000124 | exon18 | c.3689G>C | p.Arg1230Pro | HET | Benign |
| ERCC6 | NM_000124 | exon5 | c.1196G>A | p.Gly399Asp | HET | Benign |
| MEN1 | NM_000244 | exon10 | c.1636A>G | p.Thr546Ala | HOM | Benign |
| KCNJ5 | NM_000890 | exon2 | c.844C>G | p.Gln282Glu | HOM | Benign |
| WNK1 | NM_001184985 | exon1 | c.446C>T | p.Ala149Val | HET | Benign |
| WNK1 | NM_213655 | exon9 | c.2190G>C | p.Leu730Phe | HET | Benign |
| WNK1 | NM_014823 | exon11 | c.2425A>C | p.Thr809Pro | HET | Benign |
| WNK1 | NM_014823 | exon17 | c.3776G>C | p.Cys1259Ser | HOM | Benign |
| SERPINA6 | NM_001756 | exon3 | c.736T>G | p.Ser246Ala | HOM | Benign |
| SLC12A1 | NM_000338 | exon23 | c.2873T>C | p.Val958Ala | HOM | Benign |
| PLIN1 | NM_001145311 | exon5 | c.580C>G | p.Pro194Ala | HOM | Benign |
| CACNA1H | NM_001005407 | exon9 | c.1919C>T | p.Pro640Leu | HOM | Benign |

| CACNA1H | NM_001005407 | exon34 | c.6212G>A | p.Arg2071His | HOM | Benign |
| --- | --- | --- | --- | --- | --- | --- |
| PKD1 | NM_000296 | exon35 | c.10526C>T | p.Thr3509Met | HET | Benign |
| ABCC6 | NM_001171 | exon19 | c.2542A>G | p.Met848Val | HOM | Benign |
| SLC12A3 | NM_000339 | exon6 | c.791C>G | p.Ala264Gly | HOM | Benign |
| SLC12A3 | NM_000339 | exon23 | c.2738G>A | p.Arg913Gln | HET | Benign |
| NOS2 | NM_000625 | exon16 | c.1823C>T | p.Ser608Leu | HET | Benign |
| HSD17B1 | NM_000413 | exon6 | c.937G>A | p.Gly313Ser | HET | Benign |
| ATRX | NM_138270 | exon8 | c.2671C>G | p.Gln891Glu | HET | Benign |
| ALMS1 | NM_015120 | exon8 | c.1570_1571ins CTC | p.Ser524delinsSe rPro | HOM | Benign |
| PDE11A | NM_001077196 | exon17 | c.1431_1432ins TCC | p.Pro478delinsS erPro | HET | Benign |
| SLC37A4 | NM_001164279 | exon5 | c.308+1G>- | - | HOM | Benign |
| WNK1 | NM_213655 | exon9 | c.2220dupC | p.Leu740fs | HOM | Benign |
