# Supplementary material for: Recurrence of Primary Aldosteronism 10 Years After Left Adrenalectomy for Aldosterone-Producing Adenoma: A Case Report
Source: Front Endocrinol (Lausanne). 2021 Sep 24;12:728595. doi: 10.3389/fendo.2021.728595 (PMC8498213; doi:10.3389/fendo.2021.728595)

File: E02_K10KCNJ5_05.ab1 Run Started: Mar 15, 2018, 22:51:40 Signal G:670 A:444 T:864 C:991

Sample: K10KCNJ5 Lane: 5 Base spacing 10.74 157 bases in 1896 scans

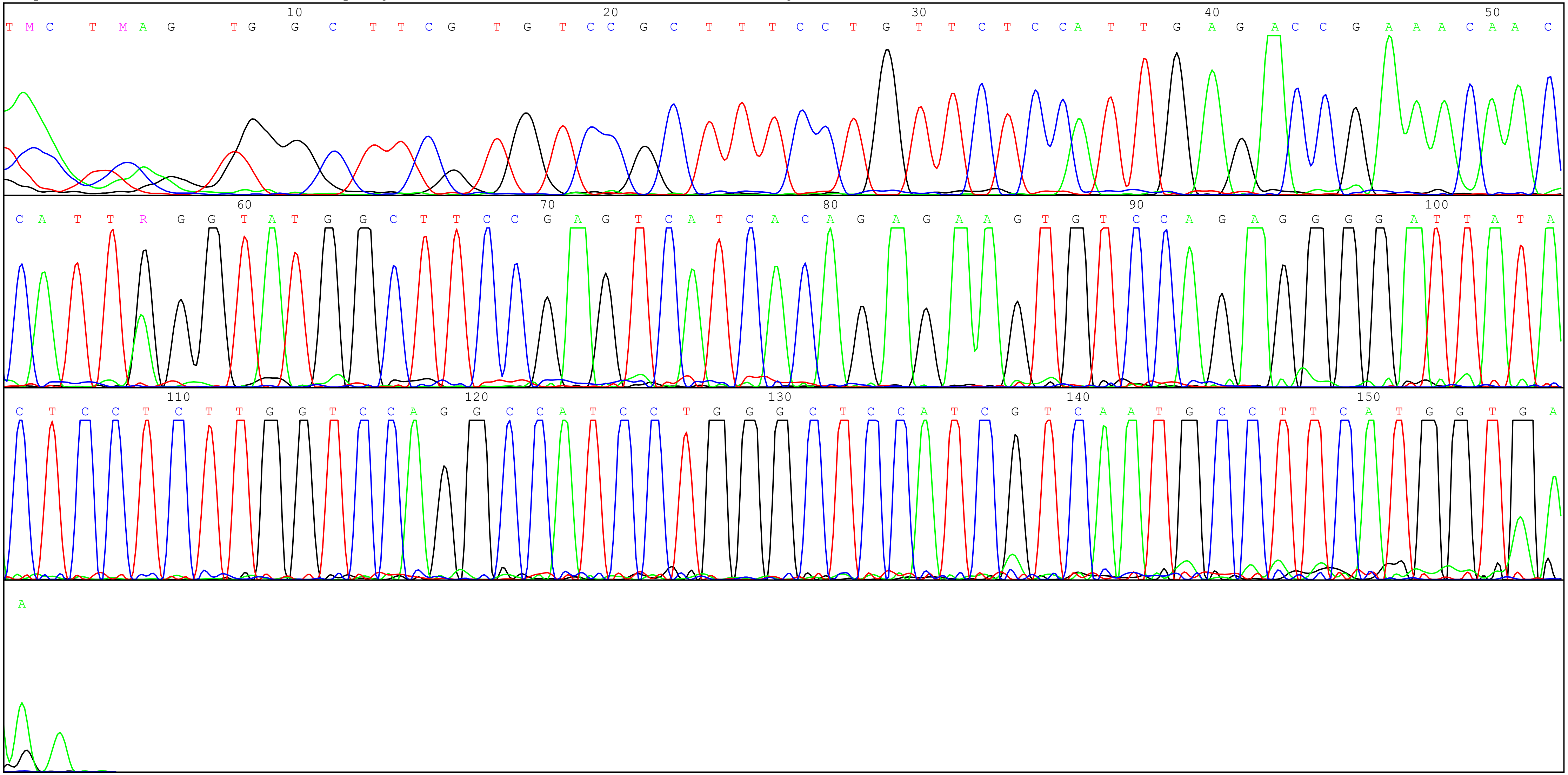

Supplement: Supplementary file 2 [file DataSheet_2.pdf]
